# Evaluation of the Venus P-valve for transcatheter pulmonary valve replacement in patients with right ventricular outflow tract dysfunction

**DOI:** 10.1080/14796678.2026.2628689

**Published:** 2026-03-10

**Authors:** Baker M. Ayyash, Ziyad M. Hijazi

**Affiliations:** Department of Cardiovascular Diseases, Sidra Medicine, Doha, Qatar

**Keywords:** Transcatheter pulmonary valve replacement (tPVR), Venus P-valve, pulmonary valve replacement, right ventricular outflow tract (RVOT), tetralogy of fallot (TOF), self-expanding valve, congenital heart disease

## Abstract

Right-ventricular outflow tract dysfunction is a common late sequela of congenital heart disease, particularly after tetralogy of Fallot repair. Balloon-expandable valves such as Melody and Sapien have transformed care for conduits and bioprostheses, but until recently most patients with large or irregular outflow tracts were not candidates for percutaneous therapy. This review appraises the Venus P-Valve, the first self-expanding transcatheter pulmonary valve for large right-ventricular outflow tracts, summarizing evidence on efficacy, safety, and regulatory status. We performed a narrative review of feasibility studies, multicenter registries, national databases, and comparative analyses reporting outcomes with this device. More than 600 implantations have been reported worldwide. Procedural success consistently exceeds 95%, with immediate restoration of pulmonary valve competence, low residual gradients, and right-ventricular reverse remodeling within 6–12 months. Follow-up to five years demonstrates durable performance and a low need for reintervention. Comparative studies suggest hemodynamic outcomes similar to surgical pulmonary valve replacement, with shorter hospitalization and lower morbidity in appropriately selected patients; pediatric series confirm feasibility with sustained benefit. Reported adverse events have been infrequent. The Venus P-Valve expands transcatheter options for large native or patched outflow tracts and currently holds CE marking in Europe and NMPA approval in China.

## Plain Language Summary

A catheter-based heart valve option for patients with a damaged pulmonary valve

The pulmonary valve helps move blood from the right side of the heart to the lungs. In people born with heart defects, particularly tetralogy of Fallot, this valve or the pathway leading to it is often damaged. Even after childhood surgery, the valve may begin to leak or become narrow again. Over time, this can cause the right side of the heart to enlarge and weaken.

For many years, treatment required repeat open-heart surgeries. While effective, surgical valves do not last forever, meaning patients often need multiple operations over their lifetime.

In recent years, doctors have been able to replace pulmonary valves using a catheter inserted through a blood vessel, avoiding open-heart surgery. Some catheter-based valves work well when the valve opening is small and regular, but many patients with tetralogy of Fallot have a much wider and uneven pathway, making these valves unsuitable.

The Venus P-valve was developed specifically for these patients. It is designed to fit larger and irregular valve pathways. Studies from multiple centers show that the valve can be implanted successfully in most patients, quickly reduces valve leakage, and allows the right side of the heart to recover. Since the first use in 2013, more than 3200 patients worldwide have been treated according to manufacturer-reported data. Follow-up of up to five years shows the valve continues to work well.

Problems after implantation are uncommon. When they occur, they may include infection, temporary heart rhythm changes, or changes seen on scans that usually do not affect valve function. The Venus P-valve has also been used successfully in selected children and teenagers.

## 1. Introduction

Right ventricular outflow tract (RVOT) dysfunction is a common long-term sequela of congenital heart disease, particularly following repair of tetralogy of Fallot (TOF). Chronic pulmonary regurgitation leads to progressive right ventricular (RV) dilation, impaired exercise tolerance, arrhythmias, and an increased risk of sudden cardiac death. Surgical pulmonary valve replacement (sPVR) has long been the standard of care; however, prosthetic valves and conduits inevitably degenerate, resulting in multiple reoperations over a patient’s lifetime [1–4]. In children, valve replacement is further complicated by somatic growth, prosthesis size limitations, and the potential need for anticoagulation [5].

The origins of this disease burden often trace back to early life. Advances in neonatal and infant palliation – such as systemic-to-pulmonary shunts and RVOT stenting – have markedly improved survival but frequently produce large, irregular RVOT anatomies due to disruption of the native annulus and patch augmentation during repair [6,7]. These complex geometries are difficult to address with conventional surgical conduits, highlighting the limitations of earlier transcatheter valve designs.

Transcatheter pulmonary valve replacement (tPVR) has transformed the management of conduit and prosthetic valve dysfunction [8–10]. The Melody valve, introduced in 2000, and subsequent balloon-expandable systems such as the Edwards Sapien 3 have demonstrated excellent mid- and long-term outcomes in patients with dysfunctional homografts or bioprosthetic valves, with procedural success rates exceeding 95% in large series [8–10]. Despite these successes, their use is confined to relatively small and uniform RVOTs, which excludes most patients with native or patched anatomies after TOF repair [8–10]. This limitation prompted the development of self-expanding alternatives designed to accommodate the large and irregular outflow tracts encountered in this population.

The Venus P-valve was developed to address this need. As a self-expanding nitinol system, it conforms to the variable geometries of large, patched, or native RVOTs and has been evaluated in multiple feasibility studies and multicenter registries across diverse patient populations [11–16]. Since the first-in-human implantation in 2013, more than 600 patients have been reported across published feasibility studies, multicenter trials, national registries, and comparative analyses with sPVR, demonstrating procedural success above 95% and sustained valve competence in mid-term follow-up [11–16].

Recent international guidelines increasingly emphasize timely pulmonary valve intervention and endorse transcatheter approaches when anatomically feasible [17]. The 2018 AHA/ACC and 2020 ESC guidelines for adult congenital heart disease recommend proactive PVR in patients with symptomatic pulmonary regurgitation and significant RV dilation to prevent irreversible dysfunction [17,18]. The 2020 ACC/AHA valvular heart disease guidelines further supports tPVR as a reasonable alternative to surgery in suitable patients [19]. Most recently, the 2024 AHA scientific statement on repaired TOF highlighted the expanding role of self-expanding transcatheter valves in long-term management strategies [20].

Collectively, these advances underscore the ongoing need for durable, safe, and anatomically versatile transcatheter solutions for patients with large RVOTs. The Venus P-valve represents a significant advance in this field, and its growing body of evidence provides the foundation for the present evaluation.

## 2. Overview of the field

Surgical pulmonary valve replacement (sPVR) has long been the standard treatment for patients with RVOT dysfunction following congenital heart disease repair [17,18]. Homografts, xenografts, and, less commonly, mechanical prostheses have been used, but their long-term durability remains modest, particularly in younger patients [1,5,21]. Conduit deterioration – driven by calcification, immune-mediated degeneration, and somatic growth – typically occurs within 10–15 years, often necessitating multiple reoperations over a patient’s lifetime [3,4,22,23]. Predictors of early conduit failure include younger age at implantation, smaller conduit diameter, and conduit type differences (homograft vs xenograft) [24–26].

The advent of tPVR has transformed this landscape by offering a minimally invasive alternative to repeat surgery [8–16]. Since its introduction in the early 2000s, tPVR has evolved from treating conduit dysfunction to addressing a broader range of anatomies, including large native RVOTs [8–16]. Among the various devices developed, self-expanding valves such as the Venus P-valve have expanded therapeutic eligibility, particularly for patients with complex or dilated outflow tracts [11–16]. These advances have redefined long-term management strategies for patients with repaired congenital heart disease, shifting the focus toward percutaneous solutions that combine procedural safety, valve durability, and quality of life [13–20].

Balloon-expandable valves, such as the Edwards Sapien 3, have expanded transcatheter treatment options to include dysfunctional bioprosthetic valves and conduits, demonstrating favorable outcomes in multicenter studies and international registries [27]. This device incorporates a bovine pericardial valve mounted on a cobalt – chromium frame, providing excellent radial strength but requiring a rigid, cylindrical landing zone for secure deployment [28,29]. As a result, its applicability remains limited in most patients with repaired tetralogy of Fallot (TOF), whose RVOTs are often large, compliant, and irregularly shaped [30]. These anatomical constraints, shared across other balloon-expandable systems, prompted the development of new self-expanding transcatheter valves designed to conform to complex RVOT geometries.

Several such systems have since been introduced. The Harmony valve (Medtronic, USA) demonstrated feasibility for large native RVOTs and recently gained approval from the U.S. Food and Drug Administration (FDA) following pivotal and pooled trial results showing favorable early and mid-term hemodynamic performance [31]. The Pulsta valve (TaeWoong Medical, Korea) has demonstrated encouraging early and mid-term outcomes in small to moderate native RVOT cohorts across Asian and European centers [32–35].

The Alterra adaptive pre-stent (Edwards Lifesciences, USA) offers an alternative strategy by creating a uniform landing zone for subsequent Sapien 3 implantation [29]. In parallel, the Myval system (Meril Life Sciences, India) has been adapted for pulmonary use, with early multicenter experiences confirming its safety and mid-term efficacy [36,37].

Among the self-expanding systems, the Venus P-Valve (Venus Medtech, China) has been evaluated in multiple feasibility studies, multicenter cohorts, and registries involving patients with large or irregular RVOTs. Its clinical experience illustrates the continuing evolution of transcatheter strategies for RVOT dysfunction, reflecting the broader movement in congenital cardiology toward less invasive, anatomy-tailored interventions [11–16].

## 3. Introduction to the device

### 3.1. Design features

**Figure 1.**
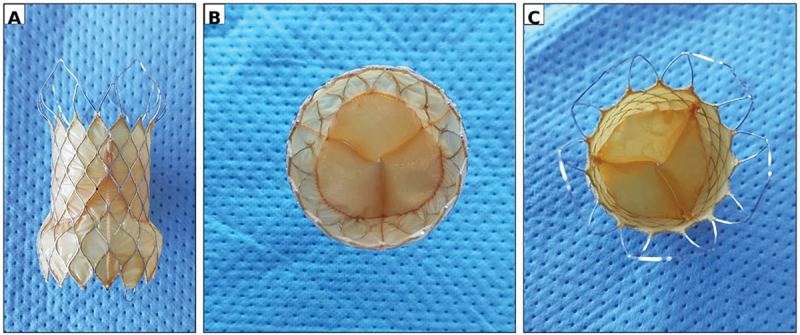
Venus P-Valve morphology and structural features.

The Venus P-Valve (Venus Medtech, Hangzhou, China) is a self-expanding transcatheter pulmonary valve system intended for patients with large or irregular right ventricular outflow tracts (RVOTs). Unlike balloon-expandable valves that require a rigid landing zone, the Venus P-Valve employs a nitinol self-expanding frame that conforms to the patient’s native anatomy, providing secure anchorage in dilated or patch-repaired RVOTs commonly seen after tetralogy of Fallot (TOF) repair (Figure 1) [11–13].

(A) Lateral profile of the Venus P-Valve showing the distal non-covered open flare, central waist housing the tri-leaflet porcine pericardial valve, and proximal covered flare/skirt designed for right ventricular outflow tract (RVOT) sealing within a nitinol diamond-shaped frame. (B) Proximal (RVOT/caudal) end view demonstrating the covered inflow flare forming the sealing skirt and the circular valve orifice centered within the waist with circumferential suture points. (C) Distal (pulmonary/cranial) end view showing symmetric leaflet coaptation with evenly spaced commissures; the non-covered distal flare preserves branch pulmonary artery flow.

Abbreviations: RVOT, right ventricular outflow tract.

### 3.2. Valve structure

The stent frame is laser-cut from nitinol and covered with porcine pericardial tissue. Three porcine pericardial leaflets are sutured within the central portion of the frame to create the functional valve. Both inflow and outflow ends are flared, a configuration that enhances stability and minimizes migration while maintaining unobstructed flow into the pulmonary arteries. The central waist houses the valve leaflets, positioned to reduce deformation and paravalvular regurgitation after deployment [11,38,39].

### 3.3. Sizing and imaging assessment

CE approved valve diameters range from 28 mm to 36 mm, with lengths ranging from 25–30 mm (the straight tubular part), allowing adaptation to a wide range of RVOT morphologies [11,13,39]. Pre-procedural computed tomography (CT) and angiography are essential for device selection, emphasizing RVOT dimensions, geometry, and proximity to the branch pulmonary and coronary arteries. Coronary compression testing during balloon sizing remains mandatory in all cases, and 3-D reconstruction is increasingly used to delineate RVOT and pulmonary artery anatomy [13,14].

### 3.4. Delivery system and implantation

The valve is delivered through a 22–24 Fr system via the femoral vein under fluoroscopic guidance. Deployment is staged and controlled, enabling precise alignment with the pulmonary annulus and branch arteries. Once released, the self-expanding frame anchors within the RVOT, the flared ends secure position, and pulmonary competence is restored immediately. Challenges remain, including the need for large-bore venous access and limited options for device retrieval once fully deployed [11–13,39].

### 3.5. Implantation steps

**Figure 2.**
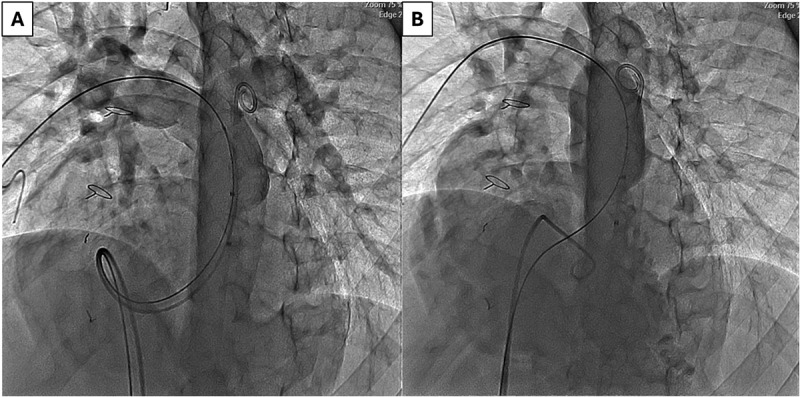
Pre-implant angiographic assessment and balloon sizing. (A) Frontal main pulmonary artery (MPA) angiogram demonstrating a markedly dilated native RVOT with free pulmonary regurgitation and unobstructed branch pulmonary arteries. (B) Frontal angiographic view during compliant balloon sizing of the native RVOT and MPA, illustrating balloon waist formation and assessment of landing zone dimensions prior to valve selection.

Abbreviations: MPA, main pulmonary artery; RVOT, right ventricular outflow tract.

**Figure 3.**
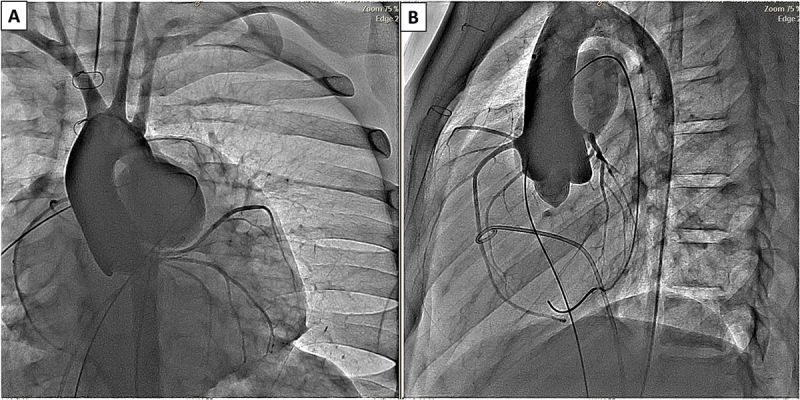
Coronary compression testing prior to Venus P-Valve implantation. (A) Frontal and (B) lateral angiographic projections obtained during simultaneous inflation of a compliant sizing balloon within the RVOT and selective coronary angiography. Coronary arteries remain patent without compression, confirming suitability for transcatheter pulmonary valve replacement.

Abbreviations: RVOT, right ventricular outflow tract.

**Figure 4.**
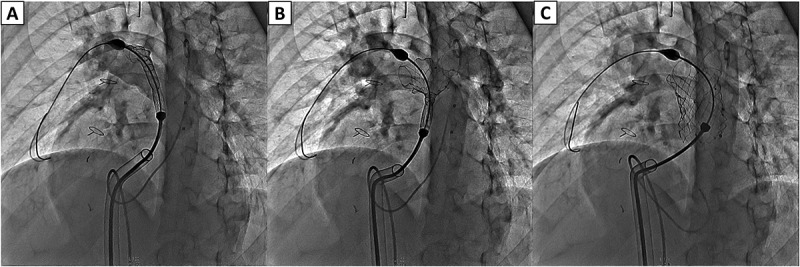
Stepwise deployment of the Venus P-Valve. Sequential lateral angiographic frames demonstrating transcatheter deployment of the Venus P-Valve within the native RVOT and MPA. (A) Partial valve release with the proximal segment constrained within the delivery sheath. (B) Continued unsheathing allows controlled self-expansion of the nitinol frame with maintenance of coaxial alignment. (C) Full deployment showing complete valve expansion, stable anchoring across the pulmonary annulus, preserved branch pulmonary artery patency, and no evidence of malposition or infolding.

Abbreviations: MPA, main pulmonary artery; RVOT, right ventricular outflow tract.

**Figure 5.**
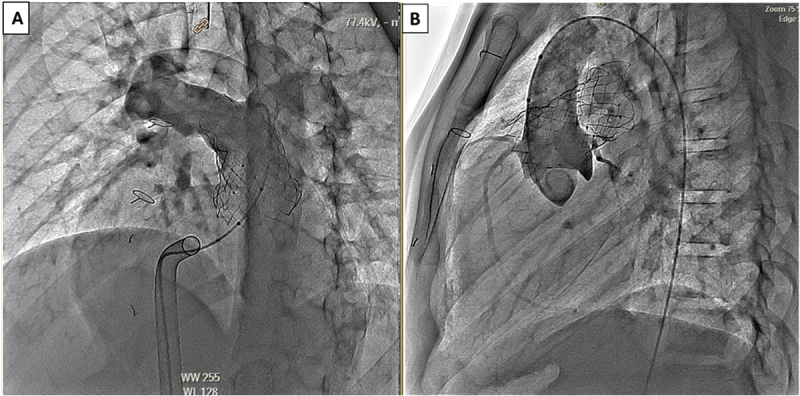
Post-implant angiographic assessment. (A) Lateral MPA angiogram following Venus P-Valve implantation demonstrating a well-expanded, coaxially aligned valve with unobstructed branch pulmonary arteries and no pulmonary regurgitation or paravalvular leak. (B) Lateral angiographic view during post-implant coronary assessment confirming preserved antegrade coronary flow without compression.

Abbreviations: MPA, main pulmonary artery.

The valve is implanted percutaneously via the femoral vein [valve also has been deployed via jugular vein in those patients with no femoral access]. The patient undergoes right and left heart diagnostic catheterization with angiography in the main pulmonary artery in 2 orthogonal views [frontal projection: either 35 degrees left anterior oblique/35 degrees cranial angulation and straight lateral. Some operators prefer right anterior oblique]. An ultra stiff guidewire is positioned in the distal right pulmonary artery posteriorly. Balloon sizing is performed inflating a large balloon in the RVOT while injecting contrast in the right ventricle. Valve size is chosen to be 2-4 mm larger than the waist. Coronary compression testing is also done in the usual fashion. The valve can be deployed either using a DrySeal sheath or without such a sheath. Accurate valve deployment is crucial to the success of the procedure ensuring both branches of the pulmonary arteries fill without any obstruction. Figures 2–5 demonstrate various steps of valve deployment.

## 4. Clinical experience

First-in-human implantation was reported in 2013 [11], followed by early feasibility series in Asia and Europe that demonstrated reproducible procedural success [12,38]. Subsequent refinements in device design and operator experience have maintained procedural success rates exceeding 95% across multicenter and national registries [13–16,39]. To date, more than 600 implantations have been reported worldwide, representing substantial international experience with a self-expanding transcatheter pulmonary valve for large or irregular RVOTs [15,16,39]. The consistency of high procedural success from early feasibility studies through contemporary multicenter registries suggests minimal impact of operator’s learning curve on early clinical outcomes.

## 5. Clinical efficacy

### 5.1. Early feasibility and short-term outcomes

In the earliest feasibility report, Cao et al. demonstrated technically successful Venus P-Valve implantation for severe pulmonary regurgitation in enlarged native RVOTs, with 100% (5/5) acute success and trace pulmonary regurgitation on early assessment [11]. Promphan et al. reproduced these findings with 100% (6/6) technical success and trace pulmonary regurgitation alongside favorable early hemodynamics [38]. In Europe, Husain et al. reported 100% (5/5) successful implants with trace pulmonary regurgitation and no major valve-related periprocedural complications [12]. Shortly thereafter, Garay et al. presented a native-RVOT series in repaired TOF (transannular patches) with 100% (10/10) acute success, early symptomatic improvement, and measurable reductions in PR fraction and right-ventricular volumes on serial imaging – establishing feasibility and early efficacy across centers and geographies [39].

At 6–12 months, multicenter and international data quantify early physiologic benefit with low residual gradients and right-ventricular reverse remodeling. In a six-center prospective cohort, Zhou et al. reported trace to mild pulmonary regurgitation at 12 months, a mean peak RVOT gradient around 16 ± 7 mmHg, and a marked RVEDVI decrease (approximately from 138 to 84 mL/m^2^) with parallel improvement in functional class – objective evidence of restored valve competence translating into chamber remodeling [13]. In complementary international experience, Morgan et al. documented sustained competence in serial CMR with significant reductions in PR fraction and indexed RV volumes at 6 and 12 months, despite surveillance detection of frame-wire findings that did not correlate with hemodynamic deterioration in reported follow-up [40]. Contemporary post-CE approval practice mirrors these signals: a single-center European series by Kramer et al. observed favorable valve function at a median 0.5 years (range 0.1–6.6) with minimal PR, low gradients, and consistent signs of RV reverse remodeling [41].

When pooled across all cohorts summarized in Table 1, Venus P-Valve implantation achieved technical success in 457 of 466 patients (98.1%). Among those successfully implanted, only two patients required reintervention during the reported follow-up period, corresponding to a reintervention rate of 0.4%.

**Table 1.** Comprehensive summary of clinical studies and registries reporting outcomes with the Venus-P Valve (2014–2025).

Author (Year) [Ref]	Study type	N	Anatomy	Tech success n/N (%)	IE n/N (%)	Frame fracture n/N (%)	VT/Arrhythmia n/N (%)	HALT n/N (%)	Migration n/N (%)	Branch PA n/N (%)	Vascular n/N (%)	Followup	Reintervention n/N (%)
Cao (2014) [11]	Feasibility case series	5	Native/patch RVOT (postTOF)	5/5 (100)	0/5 (0)	0/5 (0)	0/5 (0)	NR	0/5 (0)	0/5 (0)	0/5 (0)	3.4 mo	0/5 (0)
Husain (2016) [12]	Early European experience	5	Native RVOT (dilated)	5/5 (100)	0/5 (0)	0/5 (0)	0/5 (0)	NR	0/5 (0)	1/5 partial RPA obstruction (20)	0/5 (0)	8.5 mo median	0/5 (0)
Garay (2017) [39]	Early multicenter feasibility	10	Native RVOT (postTOF transannular patch)	10/10 (100)	0/10 (0)	0/10 (0)	0/10 (0)	NR	0/10 (0)	0/10 (0)	0/10 (0)	12 mo mean (4–21)	0/10 (0)
Long (2018) [42]	Longterm feasibility followup	14	Native RVOT (post-TOF repair)	14/14 (100)	0/14 (0)	0/14 (0)	0/14 (0)	NR	0/14 (0)	0/14 (0)	0/14 (0)	2.3 ± 0.8 y (1–3.5)	0/14 (0)
Morgan (2019) [40]	International multicenter (compassionateuse)	38	Native/dilated RVOT (postTOF)	37/38 (97)	0/37 (0)	8/30proximal flare (27)	0/37 (0)	NR	2/37 (5.4)	0/37 (0)	0/37 (0)	~25 mo	0/37 (0)
Zhou (2019) [13] & Jin (2024) [43]	Prospective multicenter cohort (1 and 5 year followup, same cohort)	55	Native/patch RVOT (postTOF)	54/55 (98.2)	5/54 (9.3)	0/54 (0)	2/54 (3.7)	NR	1/54 (1.9)	0/54 (0)	1/54 (1.9)	1 y; 5 y	1/54 (1.9)
Ou-Yang (2020) [14]	Multicenter comparison (Venus-P vs surgical)	35	Large RVOT	35/35 (100)	1/35 (2.8)	0/35 (0)	1/35 (2.8)	NR	0/35 (0)	0/35 (0)	0/35 (0)	36–48 mo	0/35 (0)
Sivakumar (2021) [44]	National registry (India, 7 centers)	29	Native/patch RVOT & conduits	27/29 (93)	1/27 (3.7)	3/27 (11.1)	1/27 (3.7)	NR	2/27 (7.4)	NR	1/29 AV fistula (3)	4 y mean	0/27 (0)
Kramer (2024) [41]	Singlecenter postCE experience	20	Large RVOT	20/20 (100)	0/20 (0)	1/20 (5)	1/20 sustained VT (5)	NR	0/20 (0)	0/20 (0)	1/20 PA perf (5)	0.5 y median (0.1–6.6 y)	0/20 (0)
Haddad (2025) [45]	Multicenter comparative (Venus-P vs SAPIEN3)	52	Native/patched RVOT (100%)	52/52 (100)	0/52 (0)	0/52 (0)	11/52 (21.1)	7/52(13.5)	0/52 (0)	1/52 LPA strut (2)	5/52 procedural/vascular AE (9.6)	Early postop	0/52 (0)
Abumehdi (2025) [46]	Pediatric cohort (single-center)	15	Native/patched RVOT	15/15 (100)	0/15 (0)	2/15 (13)	0/15 (0)	NR	1/15 (7)	1/15 RPA jailing (7)	1/15 iliac vein perf (7)	3.4 y mean	0/15 (0)
Pilati (2025) [16]	Italian registry SICPED (7 centers)	65	Native/patch RVOT	61/65 (93.8)	1/61 (1.6)	0/61 (0)	12/61 ectopy/NSVT (19.7)	4/10 (40)	0/61 (0)	0/61 (0)	1/65 (1.5)	13 mo median (1–20)	0/61 (0)
Qureshi (2025) [47]	C-Emark multicenter (European)	81	Mixed RVOT	81/81 (100)	1/81 (1.2)	0/81 (0)	1/81 sustained VT/ICD (1.2)	1/81 (1.2)	0/81 (0)	1/81 PA perf (1.2)	0/81 (0)	3 y	0/81 (0)
Kang (2025) [48]	Singlecenter retrospective (sizing/technique)	42	Native/patch RVOT	41/42 (97.6)	1/41 (2.4)	0/41 (0)	1/41 sustained VT (2.4)	NR	1/41 (2.4)	0/41 (0)	0/42 (0)	20 mo mean	1/41 (2.4)
**Pooled (total)^a^**	–	**466**	–	**457/466 (98.1)**^b^	**11/457 (2.4)**^c^	**14/457 (3.1)**^d^	**36/457 (7.9)**^e^	**12/143 (8.4)**^f^	**7/457 (1.5)**^g^	**4/457 (0.9)**^h^	**10/466 (2.1)**^i^	–	**2/457 (0.4)**^j^

Clinical studies and registries reporting procedural success and valverelated outcomes after Venus P–Valve implantation (2014–2025).

The table summarizes study design, sample size, anatomy, technical success, and major adverse events – including infective endocarditis, frame fracture, ventricular arrhythmias, hypoattenuated leaflet thickening, valve migration or embolization, branch pulmonary artery complications, vascular/accesssite events, and reinterventions – across 14 cohorts, with a final pooled row reporting aggregated numerators, denominators, and percentages for each endpoint.

Abbreviations: RVOT, right ventricular outflow tract; TOF, tetralogy of Fallot; IE, infective endocarditis; VT, ventricular tachycardia; NSVT, nonsustained ventricular tachycardia; HALT, hypoattenuated leaflet thickening; PA, pulmonary artery; RPA, right pulmonary artery; LPA, left pulmonary artery; AV, arteriovenous; ICD, implantable cardioverterdefibrillator; AE, adverse event; NR, not reported; mo, months; y, years; perf, perforation.

^a^Pooled data include 466 implantation attempts and 457 successful VenusP implants from 14 cohorts; Zhou (2019) and Jin (2024) are one cohort counted once. Denominators differ by endpoint (attempts, successful implants, or CTimaged subsets).

^b^Technical success: 457/466 (98.1%) across all implantation attempts.

^c^Infective endocarditis (IE): 11/457 (2.4%) among successfully implanted patients; heterogeneous followup, so this is a pooled proportion, not a timestandardized rate.

^d^Frame fracture: 14/457 (3.1%) among successfully implanted patients; most were localized and not associated with hemodynamic deterioration or reintervention.

^e^Ventricular arrhythmias (VT/VA): 36/457 (7.9%) among successfully implanted patients; events were predominantly early and selflimited.

^f^Hypoattenuated leaflet thickening (HALT): 12/143 (8.4%) among patients with C-Tbased surveillance only; earlier cohorts without systematic CT are excluded from this denominator.

^g^Valve migration/embolization: 7/457 (1.5%) among successfully implanted patients; events occurred early and were managed with surgical or percutaneous intervention.

^h^Branch pulmonary artery complications: 4/457 (0.9%) among successfully implanted patients; all were recognized periprocedurally or early and successfully treated.

^i^Vascular/accesssite complications: 10/466 (2.1%) among all implantation attempts; all attempts required largebore venous access and were considered at risk.

^j^Reintervention: 2/457 (0.4%) among successfully implanted patients; reinterventions were performed for hemodynamic deterioration or structural valve abnormalities.

These pooled Venus P‑Valve outcomes are broadly comparable to published data for other self‑expanding tPVR systems: Harmony TPV cohorts report technical success around 93–95% with reintervention rates of approximately 6–8% at 3–5 years, while Pulsta series describe near‑universal procedural success with very low reintervention rates in mid‑term follow‑up [31–35].

### 5.2. Mid- to long-term durability and reintervention

Five-year durability of the Venus P-Valve has now been documented. In a prospective, six-center cohort of 55 patients, Jin et al. reported 96% freedom from all-cause mortality (95% CI, 86%–99%) and 98% freedom from reintervention (95% CI, 87%–100%) at five years [43]. Formal, standardized criteria for reintervention were not prospectively defined in this or other Venus P-Valve studies; instead, reinterventions were individualized and undertaken in the presence of clinical symptoms, hemodynamic deterioration, or structural valve abnormalities such as significant regurgitation, stenosis, or device malfunction. No paravalvular leak has been detected, and only one patient developed moderate pulmonary regurgitation. Transpulmonary gradients and stent-orifice diameters remained stable compared with discharge, while right-ventricular size, left-ventricular ejection fraction, and New York Heart Association functional class all showed sustained improvement during follow-up [43]. These outcomes are consistent with the international multicenter experience outside China, which similarly demonstrated maintained valve competence and reverse-remodeling benefits into the mid-term period; surveillance-detected frame-wire findings in that cohort were not associated with hemodynamic deterioration during reported follow-up [40].

Taken together, these data outline a consistent efficacy profile for the Venus P-Valve in clinical practice. Venus P-Valve implantation is associated with high acute procedural success and minimal pulmonary regurgitation, maintains low RVOT gradients, and promotes right-ventricular reverse remodeling within the first year of follow-up. Five-year cohort data further demonstrate durable valve function and low rates of reintervention. Serial cardiac magnetic resonance (CMR) and echocardiographic assessments provide concordant evidence of sustained valve competence and chamber remodeling, while long-term data confirm stable gradients, preserved valve geometry, and persistent functional improvement [13,40].

### 5.3. Comparison with other tPVR platforms and surgery

When Venus P-Valve outcomes are viewed alongside published experience with other self-expanding tPVR systems (Harmony, Pulsta), their efficacy in large native or patched RVOTs appears broadly comparable. Across feasibility studies and multicenter cohorts, all three devices demonstrate procedural success rates exceeding 95%, reduction of pulmonary regurgitation to trivial/mild levels, low residual RVOT gradients, and early right-ventricular reverse remodeling on serial imaging. Although direct head-to-head comparisons in identical RVOT anatomies are unavailable, existing evidence indicates similar short- to mid-term hemodynamic and clinical outcomes within their intended anatomical ranges. Device choice is thus guided primarily by RVOT morphology, institutional availability, and operator experience rather than demonstrated efficacy differences [31–35]

Comparative studies indicate that, in appropriately selected patients with large native RVOTs, tPVR using the Venus P-Valve achieves hemodynamic results comparable to surgical replacement while avoiding repeat sternotomies. In a multicenter analysis, Ou-Yang et al. [14] reported that tPVR with the Venus P-Valve was a safe and effective alternative to surgery, showing lower pulmonary valve gradients at discharge and follow-up as well as improved right-ventricular and valve function at mid-term in suitable anatomies. These findings are consistent with broader tPVR experience, where cardiac magnetic resonance imaging has confirmed reductions in pulmonary regurgitant fraction and indexed RV volumes within 6–12 months, accompanied by favorable early clinical outcomes.

### 5.4. Pediatric clinical efficacy

Clinical efficacy of the Venus P-Valve in pediatric patients is increasingly supported by contemporary evidence. Abumehdi et al. reported a cohort of 15 pediatric patients (mean age 14 years; weight range 34–98.5 kg) with a 100% implantation success rate and MRI-confirmed right ventricular remodeling, demonstrated by a reduction in indexed right ventricular end-diastolic volume (RVEDVi) from approximately 158 to 118 mL/m^2^ and pulmonary regurgitant fraction from 44% to 3.6% over a mean follow-up of 3.4 years. Complications were infrequent and effectively managed, including one case of proximal valve migration requiring surgical intervention, one instance of right pulmonary artery jailing resolved by stenting, and one iliac vein perforation treated with a covered stent. No cases of infective endocarditis, clinically significant arrhythmias, or functional impairment from isolated single-strut fractures were observed. Comparable early European reports in smaller pediatric cohorts have corroborated these findings, demonstrating consistent procedural success, favorable hemodynamic improvements, and evidence of right ventricular reverse remodeling [46]. Collectively, these data substantiate the Venus P-Valve as a feasible and effective transcatheter solution for selected adolescents with large patched or native right ventricular outflow tracts, providing a durable alternative to repeat surgical pulmonary valve replacement.

## 6. Real-world evidence

Beyond controlled clinical studies, accumulating real-world experience confirms that Venus P-Valve implantation achieves comparable outcomes in everyday clinical practice. In an international multicenter cohort, Morgan et al. [40] achieved 37 successful implantations among 38 attempts, with low acute complication rates and sustained valve competence at 6–12 months. Serial CMR demonstrated significant reductions in pulmonary regurgitant fraction and indexed right-ventricular (RV) volumes, while surveillance imaging revealed frame-wire changes that were not associated with hemodynamic deterioration during follow-up. Similarly, contemporary post-CE practice at a single European center documented 100% procedural success, minimal regurgitation, low gradients, and consistent evidence of RV reverse remodeling over a median follow-up of 0.5 years (range 0.1–6.6 years) [41]. These real-world findings are concordant with results from the prospective six-center one-year cohort [13], supporting the reproducibility and procedural safety of Venus P-Valve implantation across diverse institutional settings.

## 7. Safety & tolerability

Venus P-Valve implantation has been associated with a generally favorable safety profile across feasibility studies, multicenter cohorts, and national registries encompassing more than 600 reported cases. Periprocedural mortality is low, major adverse events are infrequent, and most late complications occur within the first year after implantation. As summarized below by complication type, reported adverse events include stent frame fracture, infective endocarditis, transient ventricular arrhythmias, valve migration or positional abnormalities, hypoattenuated leaflet thickening, and rare vascular access – related events, with incidence varying according to surveillance intensity and follow-up duration. Collectively, available evidence underscores the importance of meticulous pre-procedural planning, appropriate antithrombotic strategies, strict infective endocarditis prophylaxis, and structured post-implant monitoring (Table 1).

In the pooled analysis of 457 successfully implanted patients, infective endocarditis occurred in 2.4% (11/457), frame fractures in 3.1% (14/457), ventricular arrhythmias in 7.9% (36/457), valve migration or embolization in 1.5% (7/457), branch pulmonary artery complications in 0.9% (4/457), and reintervention in 0.4% (2/457) during reported follow-up. Vascular or access-site complications were observed in 2.1% (10/466) of all implantation attempts. Among patients from cohorts undergoing CT-based surveillance, hypoattenuated leaflet thickening was identified in 8.4% (12/143) and was typically not associated with hemodynamic compromise.

By comparison, published Harmony transcatheter pulmonary valve studies report infective endocarditis rates of approximately 2–3% at 3–5 years of follow-up, major stent fracture in roughly 1–1.5%, valve migration in 3–5%, and reintervention rates in the range of 6–8% [31]. In contrast, Pulsta valve series published to date have not reported stent fracture or infective endocarditis; however, these observations are derived from smaller cohorts with shorter follow-up durations, limiting definitive conclusions regarding long-term safety and durability [32–35].

### 7.1. Stent frame fracture

Stent frame fracture has been reported in selected Venus P-Valve cohorts, with incidence varying according to study design and surveillance intensity. In the international multicenter experience reported by Morgan et al., systematic fluoroscopic follow-up identified proximal flare fractures in 27% (8/30) of evaluable patients, without associated valve dysfunction, hemodynamic deterioration, or need for reintervention over a median follow-up of approximately 25 months [40].

Lower rates of localized fractures have been described in other cohorts. In the Indian multicenter registry, Sivakumar et al. reported non-significant wire-frame fractures in 3 of 29 patients (10.3%) [44]. In a European post-CE single-center experience, Kramer et al. identified a single-strut fracture in 1 of 20 patients (5%), while a pediatric series by Abumehdi et al. reported isolated single-strut fractures in 2 of 15 patients (13.3%) during a mean follow-up of 3.4 years; none were associated with valve dysfunction, arrhythmias, or clinical impairment [41,46].

Overall, reported frame fractures – most commonly involving the proximal flared segment – have not been shown to correlate with structural valve failure or adverse clinical outcomes, supporting continued imaging surveillance rather than preemptive intervention when valve performance remains preserved.

### 7.2. Infective endocarditis

Infective endocarditis (IE) represents the most clinically significant late complication reported after Venus P-Valve implantation, with incidence varying across cohorts and follow-up duration. In the largest prospective dataset, Zhou et al. reported IE in 7.3% (4/55) of patients within the first year, including one IE-related death [13]. Extended follow-up from the same cohort by Jin et al. demonstrated a cumulative IE incidence of 9% (5/55) at five years – approximately 1% per patient-year – with outcomes including one death and one surgical reintervention, while the remaining cases were successfully managed with antibiotic therapy [43].

Lower event rates have been observed in European and registry-based experiences. The Italian SICPED registry reported IE in 1.5% (1/65) of patients at approximately 12 months, and the international CE-mark multicenter study by Qureshi et al. documented a single IE episode at 11 months, treated medically with preserved valve function [16,47]. Isolated early cases have also been described, including a report by Wang et al. of IE occurring two months post-implantation that resolved completely with targeted antibiotic therapy [49]. Notably, in the Italian registry, one IE case occurred in a patient with prior computed-tomography – detected hypoattenuated leaflet thickening (HALT), suggesting a possible – but unproven – association [16].

Collectively, available evidence indicates a non-negligible yet heterogeneous risk of IE following Venus P-Valve implantation, with events clustering predominantly within the first year. These findings reinforce the importance of strict antibiotic prophylaxis, meticulous dental hygiene, early evaluation of febrile illness, and structured post-implant surveillance.

### 7.3. Ventricular arrhythmias

Ventricular arrhythmias following Venus P-Valve implantation have been reported predominantly as transient, early events. In a recent multicenter comparative study by Haddad et al. (2025; n = 145), directly comparing self-expanding Venus P-Valve (n = 52) with balloon-expandable SAPIEN-3 (n = 93) in native or patched RVOTs, procedural success was 100% in both groups and major complication rates were comparable (9.6%, 5/52, in the Venus-P cohort). Transient ventricular arrhythmias occurred more frequently after Venus P-Valve implantation (21.1%, 11/52) compared with SAPIEN-3 (3.2%, 3/93), but all events resolved without catheter ablation or long-term antiarrhythmic therapy, and no sustained ventricular tachycardia or arrhythmic death was reported [45]. Across additional multicenter and registry experiences, sustained ventricular arrhythmias have been uncommon. In the Italian SICPED Registry, transient ventricular ectopy – occasionally short non-sustained ventricular tachycardia – was observed in 19% (12/65) of patients, typically resolving within one month on β-blocker therapy, with no cases of high-grade atrioventricular block, pacemaker implantation, or sudden arrhythmic death at a median 13-month follow-up [16]. In the CE-mark study by Qureshi et al., one patient developed sustained ventricular tachycardia requiring implantable cardioverter-defibrillator implantation and catheter ablation, without a broader signal of malignant ventricular arrhythmia across the 81-patient cohort [47]. Similar findings have been reported in pediatric populations, where Veillette et al. observed transient ventricular ectopy among Venus P-Valve recipients, while more serious arrhythmic events occurred only after Melody valve implantation [50]. Overall, available evidence indicates that ventricular arrhythmias associated with Venus P-Valve implantation are primarily early and self-limited, supporting routine rhythm surveillance – particularly in the peri-procedural period – without evidence of increased late arrhythmic risk.

### 7.4. Valve migration and positional complications

Valve migration or embolization after Venus P-Valve implantation has been reported infrequently and is largely related to procedural and anatomical factors. In the international multicenter experience reported by Morgan et al., migration occurred in 2 of 38 patients (5.3%), with one case requiring surgical stabilization and another associated with moderate tricuspid regurgitation due to device interference, without persistent hemodynamic compromise [40]. Similarly, the Indian multicenter registry (Sivakumar et al.) reported migration in 2 of 29 patients (6.9%), including one requiring surgical retrieval, highlighting the role of accurate sizing, complete device detachment, and controlled deployment [44].

Branch pulmonary artery (PA) compromise and other positional abnormalities have been described less commonly. In a pediatric cohort (Abumehdi et al., n = 15), one proximal migration (6.7%) required surgical repositioning and one right PA jailing (6.7%) was successfully treated with percutaneous stenting [46]. Additional isolated events include a case of valve infolding (Riahi et al.) requiring individualized management and a single late distal-flare thrombus reported in the CE-mark multicenter experience (Qureshi et al.), which resolved with anticoagulation and preserved valve hemodynamics [47,51].

Collectively, migration, branch PA compromise, infolding, and distal-flare thrombus are uncommon but clinically relevant events that appear closely linked to RVOT anatomy and deployment mechanics, underscoring the importance of meticulous pre-procedural imaging, balloon-based sizing, attention to distal flare – PA relationships, and structured post-implant imaging surveillance.

### 7.5. Hypoattenuated leaflet thickening (HALT)

Leaflet thrombosis and hypoattenuated leaflet thickening (HALT) have emerged as recognized post-implant imaging findings after Venus P-Valve implantation. HALT – defined as hypoattenuated leaflet thickening with or without reduced leaflet motion on computed tomography – reflects thrombotic deposition and is often subclinical, occurring without symptoms or hemodynamic compromise. The first Venus P-Valve case reported by Le Gloan et al. demonstrated complete resolution with anticoagulation, supporting a reversible thrombotic mechanism [52].

Subsequent studies have identified HALT in a subset of patients undergoing CT surveillance. In the Italian SICPED registry (Pilati et al., n = 65), CT surveillance was performed in a 10‑patient subset, and HALT was detected in 4 of these patients (40%), with preserved valve function and stable gradients; notably, one patient later developed infective endocarditis (Enterococcus), suggesting a possible association without establishing causality [16]. In a multicenter series by Haddad et al. (2025; n = 52), HALT was reported in 7 patients, including 4 with reduced leaflet mobility, without associated hemodynamic deterioration [45]. Additional insight into the prevalence and early functional implications of HALT was provided by Zhu et al., who performed systematic computed-tomography surveillance in 64 Venus P-Valve recipients within the first year after implantation. HALT was detected in 56% of patients, with reduced leaflet motion observed in 20.8%. Despite preserved transvalvular hemodynamics and similar early clinical outcomes compared with patients without HALT, those with HALT demonstrated subtly lower right-ventricular systolic function at the time of CT assessment, reflected by reduced tricuspid annular plane systolic excursion. Older age and higher body mass index were independently associated with HALT, while anticoagulation was not identified as a protective factor, underscoring the largely subclinical yet prevalent nature of this finding and its potential ventricular-level implications [53]

More recently, a systematic CT-based case series by Spagnolo et al. (n = 7) identified HALT in 5 patients (71%) at 6-month follow-up, frequently involving the right cusp and associated with reduced leaflet motion, yet without valve dysfunction, thromboembolic events, or clinical deterioration; HALT regressed following initiation of oral anticoagulation [54]. Additional thrombotic manifestations have been described, including a single late distal-flare thrombus in the CE-mark multicenter experience (Qureshi et al.), which resolved with anticoagulation and preserved valve performance [47]. While short-term clinical impact has generally been limited, the long-term implications for valve durability and any relationship with infective endocarditis remain incompletely defined. Antithrombotic strategies vary across centers (most commonly dual antiplatelet therapy for 3–6 months followed by aspirin monotherapy, with anticoagulation reserved for selected cases, particularly when HALT is confirmed); in this context, structured post-implant CT surveillance – particularly during the first year – appears prudent, with antithrombotic escalation individualized according to imaging and clinical findings.

### 7.6. Vascular and access-site events

Vascular and access‑site complications following Venus P‑Valve implantation have been infrequent and primarily related to large‑bore venous access. In the Indian multicenter registry (Sivakumar et al., n = 29), a single femoral arteriovenous fistula (3.4%) was reported and managed successfully without long‑term sequelae [44]. In the pooled cohort, vascular or access‑site events occurred in 2.1% (10/466) of implantation attempts, including iliac vein perforation requiring covered stenting and occasional pulmonary artery perforations classified as procedural/vascular events, all managed without adverse impact on long‑term valve performance.

### 7.7. Rare nonvascular complications

Rare nonvascular complications following Venus P-Valve implantation have been described. Saber et al. reported a left recurrent laryngeal nerve palsy presenting with persistent hoarseness after transcatheter pulmonary valve replacement, attributed to extrinsic compression of the nerve within the aortopulmonary window in the setting of a markedly dilated right ventricular outflow tract. Vocal cord paralysis was confirmed on laryngoscopy with no recovery of cord motion at 6 months, although symptoms partially improved with conservative therapy and valve function remained satisfactory. No additional cases of recurrent laryngeal nerve injury have been reported in larger Venus P-Valve cohorts, indicating that such events are exceedingly uncommon, but this complication emphasizes the need for careful anatomical assessment and post-procedural vigilance for atypical neurologic symptoms [55].

## 8. Regulatory affairs

The Venus P-Valve received CE marking under the European Union Medical Device Regulation (MDR) on 8 April 2022, authorizing its commercial use for tPVR in patients with significant pulmonary regurgitation and enlarged native right ventricular outflow tracts (RVOTs). It holds the distinction as the first self-expanding tPVR system approved under the stringent EU MDR framework [56]. In China, the National Medical Products Administration (NMPA) granted market approval in July 2022, facilitating national commercialization for clinical tPVR applications [57]. In the United States, the Venus P-Valve remains investigational, with no FDA approval to date. The ongoing pivotal Investigational Device Exemption (IDE) trial, PROTEUS, commenced enrollment in June 2024, aiming to support FDA pre-market approval. The US Centers for Medicare & Medicaid Services (CMS) granted coverage approval for patients enrolled in PROTEUS, enhancing access during the trial period [58,59]. This review’s efficacy and safety assertions pertain exclusively to approved indications, with investigational data provided solely for context and informational purposes.

## 9. Conclusion

Transcatheter pulmonary valve replacement (tPVR) using the self-expanding Venus P-Valve extends catheter-based therapy to patients with enlarged native RVOTs who are not suitable candidates for balloon-expandable systems. Multiple sources – including feasibility studies, multicenter one-year cohorts, international registries with serial cardiac magnetic resonance imaging (CMR), and five-year follow-up data – demonstrate the device’s high acute procedural success, minimal residual pulmonary regurgitation, low RVOT transvalvular gradients, and consistent right ventricular reverse remodeling. Signals of mid-term durability include stable valve geometry, sustained functional improvement, and low rates of reintervention. The safety profile is favorable: complications are uncommon and generally manageable, with infective endocarditis (IE) representing the principal serious late event, predominantly occurring within the first-year post-implantation. Reported frame-wire fractures have not been associated with hemodynamic deterioration during follow-up. Emerging observations – such as hypoattenuated leaflet thickening (HALT), rare valve migration, branch pulmonary artery complications, and early transient ectopy – highlight the critical role of structured imaging surveillance and strict adherence to IE prophylaxis protocols. In appropriately selected anatomies, the Venus P-Valve offers a minimally invasive alternative to surgical pulmonary valve replacement, with physiological outcomes that are comparable in the short term and encouraging through five years. Ongoing clinical trials and registries will further refine patient selection criteria, antithrombotic strategies, and long-term device durability. No device-specific studies have evaluated the cost-effectiveness or quality-of-life impact of the Venus P-Valve, and similar data are currently lacking for other self-expanding tPVR systems; thus, economic and patient-reported outcomes remain important areas for future research.

## Article highlights

- The Venus P-valve is the first self-expanding transcatheter pulmonary valve designed specifically for large or irregular right ventricular outflow tracts (RVOTs), addressing significant limitations of balloon-expandable systems.
- Over 600 implantations have been reported in published studies, with total worldwide experience exceeding 3000 cases, procedural success rates consistently above 95%, immediate restoration of valve function, and sustained right ventricular reverse remodeling documented up to five years.
- Mid-term durability is supported by stable valve geometry, functional improvements, low reintervention rates, and no significant structural degeneration observed in serial imaging studies.
- Safety profile is favorable, with low complication rates; infective endocarditis (IE) is the principal serious late adverse event, primarily occurring within the first year after implantation.
- Emerging findings include hypoattenuated leaflet thickening (HALT), rare valve migrations, branch pulmonary artery events, and transient rhythm disturbances, highlighting the importance of structured imaging and preventive care peri- and post-implantation.
- Pediatric use has demonstrated feasibility and efficacy, with documented improvements in right ventricular volumes and pulmonary regurgitation fraction in adolescent cohorts, expanding therapeutic options beyond surgical valve replacement.
- Regulatory approvals include CE marking in Europe and approval by the National Medical Products Administration (NMPA) in China; in the United States, the Venus P-Valve remains investigational, with safety and efficacy currently being evaluated in an ongoing Investigational Device Exemption (IDE) trial (PROTEUS).
- The Venus P-valve represents a minimally invasive, durable alternative to surgery for selected patients with large native or patched right ventricular outflow tracts (RVOTs), with ongoing studies expected to refine patient selection, antithrombotic strategies, and long-term durability.
